# Establishment of a swine model of delayed bleeding after endoscopic procedure

**DOI:** 10.1002/deo2.411

**Published:** 2024-07-23

**Authors:** Shohei Uehara, Fumisato Sasaki, Hisashi Sahara, Akihito Tanaka, Makoto Hinokuchi, Hidehito Maeda, Shiho Arima, Shinichi Hashimoto, Shuji Kanmura, Akio Ido

**Affiliations:** ^1^ Digestive and Lifestyle Diseases Kagoshima University Graduate School of Medical and Dental Sciences Kagoshima Japan; ^2^ Division of Experimental Large Animal Research Life Science and Laboratory Animal Research Unit Center for Advanced Science Research and Promotion Kagoshima University Kagoshima Japan

**Keywords:** animal model, delayed bleeding, endoscopic resection, heparin, miniature swine

## Abstract

**Objectives:**

Although delayed bleeding after endoscopic procedures has become a problem, currently, there are no appropriate animal models to validate methods for preventing it. This study aimed to establish an animal model of delayed bleeding after endoscopic procedures of the gastrointestinal tract.

**Methods:**

Activated coagulation time (ACT) was measured using blood samples drawn from a catheter inserted into the external jugular vein of swine (*n* = 7; age, 6 months; mean weight, 13.8 kg) under general anesthesia using the cut‐down method. An upper gastrointestinal endoscope was inserted orally, and 12 mucosal defects were created in the stomach by endoscopic mucosal resection using a ligating device. Hemostasis was confirmed at this time point. The heparin group (*n* = 4) received 50 units/kg of unfractionated heparin via a catheter; after confirming that the ACT was ≥200 s 10 min later, continuous heparin administration (50 units/kg/h) was started. After 24 h, an endoscope was inserted under general anesthesia to evaluate the blood volume in the stomach and the degree of blood adherence at the site of the mucosal defect.

**Results:**

Delayed bleeding was observed in three swine (75%) in the heparin‐treated group, who had a maximum ACT of >220 s before the start of continuous heparin administration. In the non‐treated group (*n* = 3), no prolonged ACT or delayed bleeding was observed at 24 h.

**Conclusion:**

An animal model of delayed bleeding after an endoscopic procedure in the gastrointestinal tract was established using a single dose of heparin and continuous heparin administration after confirming an ACT of 220 s.

## INTRODUCTION

Endoscopic submucosal dissection (ESD) and resection (EMR) are well‐established treatments for early gastric tumors.^1^ Delayed bleeding occurs in 1.8–15.6% of patients after ESD.^2^, ^3^, ^4^, ^5^, ^6^ Several studies have shown that antithrombotic agents and resection size are significant risk factors for bleeding after ESD.^2^, ^3^, ^6^ According to a predictive model based on risk stratification for causing post‐ESD ulcer bleeding (BEST‐J score) recently reported by Hatta et al., the rate of delayed bleeding is 2.8% in the low‐risk group, whereas it is 29.7% in the very‐high‐risk group.^7^ With the advent of an aging society, the number of patients receiving antithrombotic drugs is increasing,^8^, ^9^ and delayed bleeding after ESD has become a great problem. Bleeding after an endoscopic procedure can sometimes be controlled by endoscopic hemostasis by coagulation of the blood vessels with electrocautery, hemostatic forceps, or by suturing with endoclips.^10^ However, it can sometimes lead to life‐threatening conditions requiring blood transfusion, arterial embolization with vascular interventional radiology, or emergency surgery.^10^, ^11^, ^12^ The most appropriate method for preventing delayed bleeding after ESD remains unknown.^13^ Although animal models simulating gastrointestinal bleeding have been reported, they have not been widely used because of the time and cost involved and the need for a surgical approach.^14^, ^15^, ^16^ Recently, animal models of intraoperative bleeding with anticoagulants^17^ and bolus administration of heparin^18^ for endoscopic hemostasis training have been reported. However, to the best of our knowledge, there are no appropriate animal models to validate prophylaxis for delayed bleeding. This study aimed to establish an animal model of delayed bleeding after endoscopic procedures of the gastric mucosa.

## MATERIALS AND METHODS

### Experimental animals

Seven CLAWN miniature swine (age, 6 months; weight, 13–14 kg; procured from the Kagoshima Miniature Swine Research Center) were used in the present study. Miniature swine were injected intramuscularly with 15 mg/kg ketamine (Daiichi Sankyo Propharma Co., Ltd.) and 2 mg/kg xylazine (Bayer Yakuhin, Ltd.) as premedications. An endotracheal tube (Smith Medical Japan) was inserted, and anesthesia was maintained using isoflurane (DS Pharma Animal Health Co., Ltd.).^19^

An upper gastrointestinal endoscope was inserted orally into seven swine to create a mucosal defect site in the stomach using an EMR with ligation (EMR‐L) technique.^20^, ^21^ Four swine were treated with heparin, whereas three were not. Heparin was administered via a catheter inserted into the external jugular vein.

The animals were excluded from solid food consumption from the day before the experiment until the day after the experiment, with free access to water. Feeding was performed by a specialized animal experimenter at the Kagoshima University Animal Experimental Facility, and symptoms were checked daily by the experimenter. Necropsies were performed by several experimenters in accordance with the ethical guidelines of the Kagoshima University Animal Experimentation Facility. No proton pump inhibitor was administered after EMR‐L. Animal care, housing, and surgery were performed in accordance with the guidelines of the Kagoshima University Animal Experiment Committee.

### Catheter insertion

Catheters (Argyle Fukuroi, CV catheter, 14 G × 30 cm; Cardinal Health) were inserted for blood sampling and heparin administration. Under general anesthesia, the catheters were inserted into the bilateral external jugular veins using the cut‐down method.^22^ One catheter was inserted for blood collection and the other for heparin administration.

### EMR‐L procedure

**FIGURE 1:**
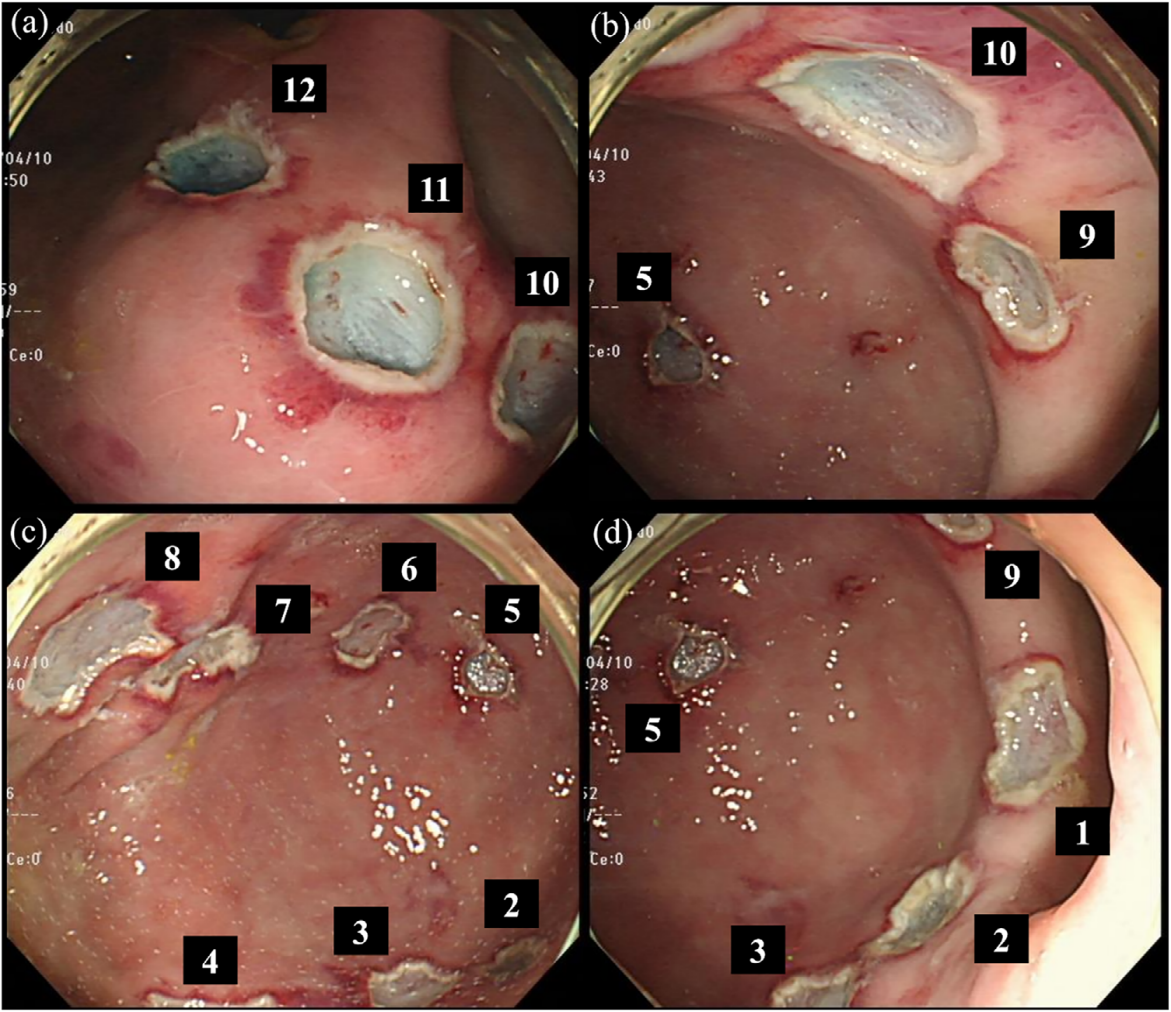
**Endoscopic findings of ulcers after endoscopic submucosal resection with ligation**. Ulcers are created using endoscopic submucosal resection with ligation at 12 locations: lesser curvature, anterior wall, and posterior wall of the upper gastric body and middle gastric body, gastric angle, and lower gastric body respectively.

Twelve artificial gastric ulcers were created by an endoscopist who performed EMR‐L using an upper gastrointestinal endoscope (GIF‐Q260J; Olympus) and a video scope system (EVIS LUCERA CV‐260SL; Olympus) to obtain reliable data on delayed bleeding. Markings were made on the lesser curvature, anterior wall, and posterior wall of the upper gastric body and middle gastric body, gastric angle, and lower gastric body respectively; using the tip of the polypectomy snare (Captivator II 15 mm; Boston Scientific) in coagulation mode, for a total of 12 locations (Figure 1). One milliliter of 0.9% saline (Otsuka Pharmaceutical Co. Ltd.) mixed with indigo carmine was injected locally using an injection needle with adequate lifting of the submucosa. Endoscopic variceal ligation (EVL) was performed using a ligating device (Pneumo Activate EVL device; Sumitomo Bakelite), followed by snaring with a polypectomy snare and pulse‐cut resection with a high‐frequency device (Pulse‐Cut Fast mode, 120 W, ESG‐100; Olympus).^20^, ^21^ All EMR‐Ls were performed by Shohei Uehara.

### Heparin administration, activated coagulation time measurement, and swine dissection

**FIGURE 2:**
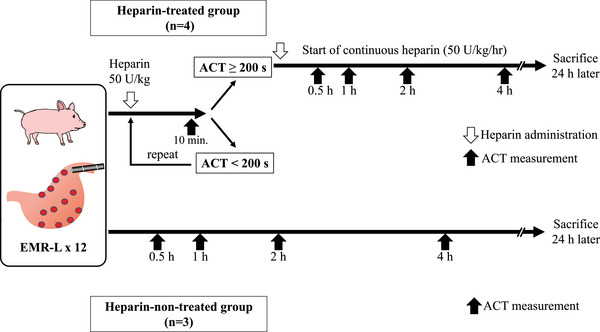
**Experimental protocol**. The protocols followed in the heparin‐treated and non‐treated groups are shown.

The experimental protocol is illustrated in Figure 2. A variety of antithrombotic drugs are used in clinical practice, including antiplatelet agents such as aspirin and anticoagulants such as warfarin and direct‐acting oral anticoagulants. Heparin was chosen for this study because it is easy to administer intravenously and can be adjusted using activated coagulation time (ACT) as an indicator. After EMR‐L, 50 units/kg of unfractionated heparin (heparin sodium; Mochida Pharmaceutical Co., Ltd.) was administered intravenously; after 10 min, ACT was measured using a coagulation analyzer (Hemochron Jr Signature+; Accriva Diagnostics, Inc.). Administration of 50 U/kg unfractionated heparin was repeated every 10 min until the ACT was >200 s. The protocol was based on previous reports.^18^ Continuous heparin administration (50 U/kg/h) was initiated after the ACT was >200 s, using a portable disposable infusion pump (SUREFUSER A, SFS‐1002D, Sustained flow 2.1 mL/h; NIPRO). We measured ACT at 0.5, 1, 2, and 4 h after starting continuous heparin administration. After 24 h, the swine was examined endoscopically to assess the blood volume in the stomach and the extent of blood adherence to mucosal defects. The animal was sacrificed at the end of the procedure by intravenous injection of thiamylal sodium (ISOZOL; Nichi‐Iko Pharmaceutical Co., Ltd.) 90 mg/kg and potassium chloride (Terumo Corporation) 20 mEq.^19^ The abdomen of the dead swine was dissected and the stomach was removed.

### Outcome measures

The primary endpoint was the presence or absence of clots in the stomach. The secondary endpoint was the number of ulcer bottoms with attached clots. The scope was inserted again 24 h after the procedure under general anesthesia, the presence of intragastric clots was confirmed by endoscopic observation, and the number of clots at the base of the ulcer was counted. The presence or absence of hematemesis on the day after the procedure was also checked, as well as the presence or absence of tar stools at autopsy. Hemoglobin levels were measured immediately after catheter insertion and between anesthesia and endoscopic observation on the day after the procedure and compared. A swine was considered to have delayed bleeding if at least one of the following conditions was met: obvious overt gastrointestinal bleeding such as hematemesis or hematemesis 24 h after the procedure and endoscopy showing a clot or blood retention in the stomach.

### Statistical analyses

The statistical significance of the difference between the two groups was calculated using the Student's t‐test or Welch's t‐test, depending on the results of the Levine test for normality and equality of variances. Statistical significance was set at *p* < 0.05. All statistical analyses were performed using IBM SPSS Statistics Base 29 software (IBM Corp.).^19^

## RESULTS

The characteristics and results for each swine are shown in Table 1. There was no significant difference in mean body weights between the heparin‐treated and non‐treated (13.9 kg vs. 13.5 kg, respectively) models. The diameter of all the ulcer beds (*n* = 12) was approximately 10 mm. The submucosal layer remained at the base of the ulcer, and the muscular layer was visible. No obvious perforations or persistent bleeding was observed.

**TABLE 1:** Characteristics and outcomes of the two distinct models.

Model No.	Body weight (kg)	Heparin administration	Continuous heparin dose	ACT at the start of continuous heparin	Max ACT	Vomiting blood	Intragastric hematoma	Intragastric blood	Blood clots in ulcers	Number of blood clots adhered	Hb before EMR‐L	Hb after EMR‐L	Tarry stool	Delayed bleeding
1	13.1	+	50 U/kg/h	227	227	+	+	+	+	1	10.6	11.6	Cecum	+
2	14.7	+	50 U/kg/h	252	252	‐	‐	+	+	3	11.7	8.8	Ileum	+
3	13.9	+	50 U/kg/h	201	201	‐	‐	‐	+	3	11.4	14.4	None	‐
4	14.0	+	50 U/kg/h	273	273	‐	+	+	+	10	11.3	9.9	Cecum	+
5	12.4	‐	‐	‐	103	‐	‐	‐	‐	0	12	13.8	None	‐
6	14.1	‐	‐	‐	110	‐	‐	‐	+	1	10.6	11.6	None	‐
7	14.1	‐	‐	‐	119	‐	‐	‐	+	1	10.9	11.5	None	‐

Abbreviations: ACT, activated clotting time; EMR‐L, endoscopic submucosal resection with ligation; Hb, hemoglobin.

**FIGURE 3:**
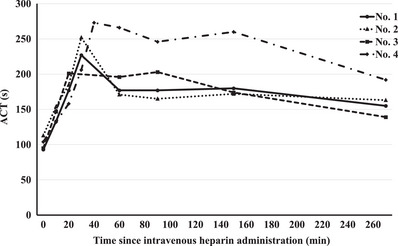
**Activated clotting time (ACT) over time in swine treated with intravenous heparin**. Heparin is administered to the first three swine until the ACT is >200 s. The maximum ACT before continuous heparin infusion is 227 s for the first swine, 252 s for the second, and 201 s for the third.

Figure 3 summarizes the changes in ACT during heparin administration. The first three swine in the heparin‐treated group were repeatedly infused with 50 U/kg heparin until the ACT was >200 s. The maximum ACT before continuous heparin infusion was 227, 252, and 201 s for the first, second, and third swine, respectively. Delayed bleeding was observed in the first and second swine but not in the third. As the ACT before continuous heparin infusion was >220 s in the first and second swine, an additional experiment was conducted with the maximum ACT before continuous heparin infusion set to >220 s to increase the incidence of delayed bleeding. The maximum ACT before the continuous heparin infusion in the fourth swine was 273 s.

**FIGURE 4:**
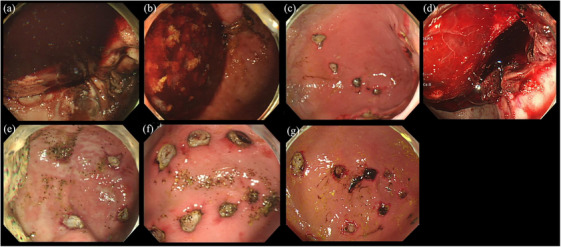
**Endoscopic findings 24 h after endoscopic submucosal resection with ligation (EMR‐L)**. Endoscopic findings after 24 h of EMR‐L are shown. (a–d) show the findings in swine that had received heparin after EMR‐L. In (a), (b), and (d), the maximum activated clotting time (ACT) before the start of continuous heparin administration is >220 s, and a large hematoma is observed in the stomach. As shown in (c), the maximum ACT before the start of continuous heparin administration is 201 s, and no delayed bleeding is observed. (e–g) do not show delayed bleeding as these swine did not receive heparin. EMR‐L, endoscopic submucosal resection with ligation.

**FIGURE 5:**
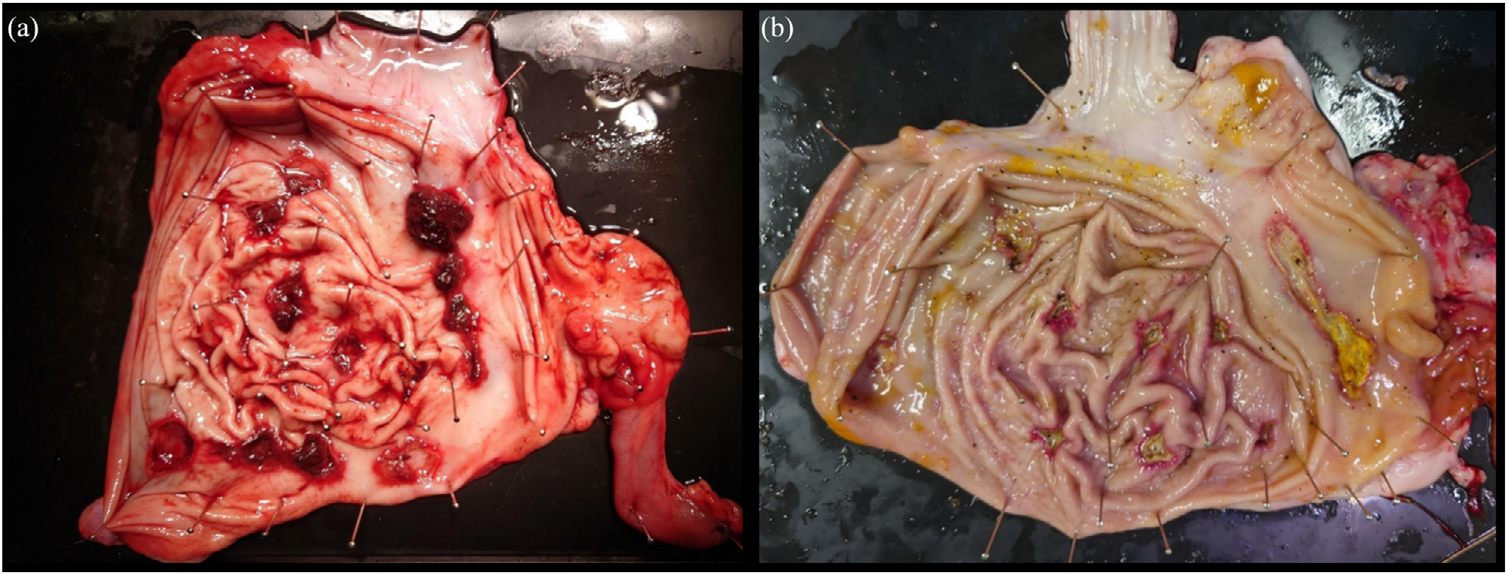
**Macro images of resected stomachs**. (a) Heparin‐treated group: 10 of the 12 ulcers show clots at the bottom. (b) Non‐treated group: no clots at the bottom of the ulcer.

The primary endpoint was the presence or absence of blood clots in the stomach. Three of the four swine (75%) in the heparin‐treated group had large intragastric blood clots; all three had maximum ACT >220 s before the start of continuous heparin administration. In the non‐treated group (*n* = 3), there was no prolonged ACT or intragastric blood clot formation after 24 h (Figure 4). Macroscopic observation of the resected stomach showed clots at the bottom of 10 of the 12 ulcers in the heparin‐treated group and no clots at the bottom of the ulcers in the non‐treated group (Figure 5). The secondary endpoint was the number of ulcer bottoms with attached clots. The mean number of clots adherent to the bottom of the ulcer in swine with a maximum ACT of ≥220 s before the start of continuous heparin administration (*n* = 3) and in the heparin non‐treated group (*n* = 3) was not significantly different (4.7 and 0.7, respectively; *p* = 0.279), but there was a trend for more clots in the heparin‐treated group.

The site of bleeding was the anterior wall of the upper gastric body (*n* = 4), the middle gastric body (*n* = 2), the anterior wall of the middle gastric body (*n* = 2), and the middle and lower gastric body (*n* = 2) of the four animals in the heparin‐treated group, and the anterior wall of the lower gastric body (*n* = 2) of the three animals in the heparin‐non‐treated group.

The ulcer bases were all approximately 10 mm in diameter, and the rate of postoperative bleeding did not differ according to ulcer size.

Two of the three pigs with delayed hemorrhage had decreased hemoglobin levels and tarry stools in the ileum and cecum after 1 day. In contrast, all the pigs without delayed hemorrhage had increased hemoglobin levels and no tarry stools after 1 day.

## DISCUSSION

In the present study, we established an animal model of delayed bleeding by administering heparin to miniature swine following an endoscopic procedure of the gastric mucosa. To the best of our knowledge, this is the first animal model for delayed bleeding after endoscopic procedures. The establishment of an animal model of delayed bleeding after an endoscopic procedure is crucial for validating methods and new techniques for preventing delayed bleeding.

The swine is the most accepted surgical model because its physiological and anatomical characteristics are similar to those of humans.^14^, ^15^, ^17^ The primary biological function of heparin is to inhibit FIIa and FXa in the coagulation cascade, and it has been used in various settings. Heparin can be administered intravenously (bolus or continuous) or subcutaneously.^23^, ^24^ A recent study reported that heparin can be safely administered in animals while monitoring ACT until the ACT is >200 s.^18^ ACT can represent overall blood coagulation capacity as influenced by activated partial thromboplastin time, prothrombin time, and platelets.^25^ Therefore, we used the ACT to monitor the effects of heparin in swine. In humans, the goal should be ≥400–480 s during artificial heart‐lung management, ≥250 s during percutaneous coronary intervention, and 180–200 s for percutaneous cardiopulmonary support and intra‐aortic balloon pumping.^26^, ^27^, ^28^ Thus, 220 s is not too high and is considered reasonable from an animal safety perspective.

We used a cut‐down method that allowed the placement of a catheter in the external jugular vein and reliable administration of heparin. A portable continuous infusion pump was used to administer a defined amount of heparin for an extended period. To the best of our knowledge, no previous studies have investigated the continuous administration of heparin in swine using this method.

In this study, we chose an EMR‐L method for gastric mucosal resection. EMR‐L allows the resection of more submucosal layers than EMR,^29^ resulting in a mucosal defect similar to that in ESD. Furthermore, EMR‐L allows uniform mucosal defects to be performed in a shorter time. The reason for creating 12 mucosal defects in the stomach was that it was unclear which areas of the swine stomach were more prone to bleeding.

Our study has several limitations. First, this was a preclinical animal study involving only seven swine. Second, in clinical practice, various antithrombotic agents are used, including antiplatelet agents such as aspirin and anticoagulants such as warfarin and directly acting oral anticoagulants. However, only heparin was used in this study. In the future, postendoscopic treatment models using various antithrombotic agents should be established. Third, we confirmed the effects of heparin on ACT, although the anticoagulant effect of heparin is usually monitored using activated partial thromboplastin time.^30^ This is because ACT can represent the overall coagulability^23^ and can be measured more quickly than activated partial thromboplastin time. Fourth, EMR‐L was used in this study to induce mucosal injury. As outlined above, EMR‐L can create a uniform submucosal resection, but the method is strictly different from ESD, in which delayed bleeding is a problem, and a post‐ESD bleeding model also needs to be investigated.

In conclusion, an animal model of delayed bleeding after an endoscopic procedure in the gastrointestinal tract was established using a single dose of heparin and continuous heparin administration after confirming an ACT of 220 s. This model can potentially be used for various endoscopic procedures, simulations, and developments of new medical devices.

## CONFLICT OF INTEREST STATEMENT

None.

## ETHICS STATEMENT

Informed Consent: N/A

Registry and the Registration No. of the study/trial: N/A

Animal Studies: All experiments were approved by the Animal Care and Use Committee of Kagoshima University (Approval number: MD22039). These animal experiments conform to the institutional standards of the Committee for Animal Research of Kagoshima University.
